# Integrating emerging technologies for varicose veins: A systematic review of comparative efficacy

**DOI:** 10.12669/pjms.42.3.12513

**Published:** 2026-03

**Authors:** Danyal Ahmad, Salwa Atta, Muhammad Naveed Babur, Sahar Aslam, Syeda Khadija Kazmi, Khizra Hamid

**Affiliations:** 1 Danyal Ahmad, PPDPT, BS-PT. Assistant Professor, University of Management and Technology, Sialkot, Pakistan, Superior University, Lahore, Pakistan; 2 Salwa Atta, MS-NMPT, DPT. Assistant Professor, Faculty of Rehabilitation Sciences, Lahore University of Biological and Applied Sciences, Lahore, Pakistan, Superior University, Lahore, Pakistan; 3 Muhammad Naveed Babur, PhD. Rehabilitation Sciences, PPDPT, BS-PT Professor, Dean, Faculty of Allied Health Sciences, Lahore, Pakistan, Superior University, Lahore, Pakistan; 4 Sahar Aslam, MS-OMPT, PPDPT, BS-PT, Superior University, Lahore, Pakistan; 5 Syeda Khadija Kazmi, MS-MSK, DPT. Lecturer, Health Sciences, University of Management and Technology, Sialkot, Pakistan, Superior University, Lahore, Pakistan; 6 Khizra Hamid, MS-OMPT, DPT, Superior University, Lahore, Pakistan

**Keywords:** Emerging Technology, Physiotherapy, Pain, Quality of Life, Rehabilitation, Varicose Veins

## Abstract

**Background & objective:**

This systematic review studies the comparative effects of emerging technology-integrated physiotherapy interventions in the treatment of varicose veins, focusing enhanced venous return, relief of symptoms and improving functional outcomes. By synthesizing existing evidence, this review seeks to identify the potential benefits, limitations, and clinical implications of these integrated approaches.

**Methodology:**

The review was registered under PROSPERA CRD420251006176 and was conducted from April to May 2025. A detailed literature review was conducted through PubMed (MEDLINE), Scopus and Web of Science databases published between March 2020 to March 2024 for experimental studies. The review fulfilled PRISMA guidelines and included Randomized controlled Trials above score of seven on Pedro Scale. Intervention strategies based on emerging technologies and wearable devices were included in the review. Studies published in languages other than English were excluded. Outcome patterns were identified by conducting by qualitative analysis.

**Results:**

Technology integrated interventions have demonstrated clinical improvements in venous severity scores, pain relief and patient adherence. Few studies have considerable challenges such as sustainability concerns in long term use. These emerging technology-based physiotherapy interventions offer a promising approach to improving outcomes in patients with varicose veins.

**Recommendations:**

Future researches should exhibit long term outcomes of these interventions on patient outcomes and patient performance.

***PROSPERO Registration Number:*** PROSPERO 2025 CRD420251006176.

## INTRODUCTION

Varicose veins is a common venous disorder in adults characterized by enlarged, twisted veins affecting a significant proportion of the global population. Non ulcerated varicose veins often cause discomfort, pain, swelling and aesthetic concerns which altogether significantly affecting quality of life.^1^ Conventional physical therapy interventions such as compression therapy, exercises and manual lymphatic drainage have been few of the management techniques for non-ulcerated varicose veins.^2^ However, with the advancing years and evolution of technology, innovative tools and approaches have now enhanced the efficacy of these interventions.

Some wearable devices, telerehabilitation platforms, biofeedback systems and advanced imaging techniques are being widely used in healthcare practices. These technologies enhance the ability to diagnose accuracy, personalize treatment plans and enhance patient engagement and adherence to therapy. For instance, wearable compression devices with real-time monitoring capabilities can optimize pressure application, while tele-rehabilitation platforms enable remote supervision and support, making physiotherapy more accessible.^3^ Despite these advancements, the comparative efficacy of integrating such technologies with traditional physiotherapy interventions for non-ulcerated varicose veins remains underexplored.

This systematic review aimed to evaluate the comparative efficacy of integrating emerging technologies with conventional physiotherapy interventions for the management of non-ulcerated varicose veins. By synthesizing existing evidence, this review seeks to identify the potential benefits, limitations, and clinical implications of these integrated approaches. Furthermore, it will provide insights into how technological advancements can be harnessed to optimize patient outcomes, improve therapeutic adherence, and address the challenges associated with traditional physiotherapy methods. Ultimately, this review aims to inform clinicians, researchers, and policymakers about the role of emerging technologies in enhancing the management of non-ulcerated varicose veins and guide future research in this evolving field.

## METHODOLOGY

This systematic review examines the different interventions and their effects on varicose veins. It was conducted as per the Preferred Reporting Items for Systematic Reviews and Meta-Analyses (PRISMA) guidelines, which may include randomized controlled trials (RCTs) that compare the efficacy of integrating emerging technologies with physiotherapy interventions versus traditional physiotherapy alone.

**Fig.1:**
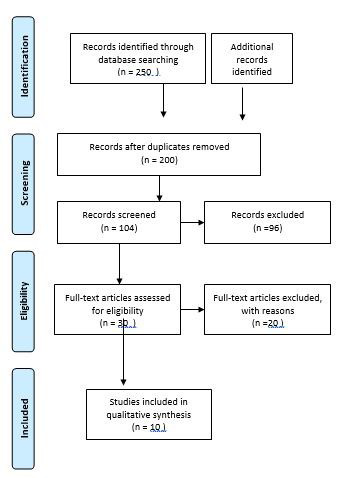
PRISMA Diagram.

### Search Strategy and Data Sources:

In March 2025, a comprehensive electronic search was performed across the databases PubMed, Scopus, Web of Science and the Cochrane Library. Our search focused on two key concepts: (1) physical therapy interventions for varicose veins and (2) integration of these into emerging technology. The search was based on both MeSH terms and keywords to ensure a complete coverage. The key terms searched included: “varicosities”, “varicose veins”, “chronic venous insufficiency”, “physiotherapy”, “exercise therapy”, “emerging technologies”, “digital health” and “tele-rehabilitation”. Boolean operators were also used to refine the search.

### Databases searched:

The main databases searched are ASSIA - Applied Social Sciences Index & Abstracts, CENTRAL - Cochrane Central Register of Controlled Trials, CINAHL - Cumulative Index to Nursing and Allied Health Literature, CLIB - The Cochrane Library, Embase.com, MEDLINE, PubMed, SCI - Science Citation Index and Scopus.

### Other important or specialist databases that were searched:

Directory of Open Access Journals (DOAJ), Europe PMC, PEDro (Physiotherapy Evidence Database), Open Science Framework (OSF), ResearchGate, PubMed Central (PMC).

### Methods of identifying studies:

Studies were identified by contacting authors or experts, looking through all the articles that cite the papers included in the review (“snowballing”), reference list checking, searching conference proceedings, searching dissertation and thesis databases and searching trial or study registers.

### Selection process:

Studies were screened independently by two people with a process to resolve differences.

### Participants and Population:

The participants are individuals diagnosed with non-ulcerated varicose veins. This may include adults of varying ages, genders, and severity of the condition, but specifically excludes those with ulcerated varicose veins.

### Interventions:

The interventions include physiotherapy interventions that are integrated with emerging technologies. Emerging technologies could encompass a range of interventions including Physiotherapy; Biomedical Device; Telerehabilitation; Laser therapy; Ultrasound therapy; Compression Therapy; Physical Exercises; Manual Therapy; Intermittent Pneumatic Compression Therapy; Pneumatic Compression Therapy; Neuromuscular Stimulation; Biofeedback Procedure

### Comparisons:

The comparison groups involve traditional physiotherapy interventions without the integration of emerging technologies. This could include standard care practices such as compression stockings, exercise programs, or manual lymphatic drainage or no treatment.

### Outcomes:

The primary outcomes are likely to include measures of comparative efficacy, such as improvement in symptoms (e.g., pain, swelling, heaviness), quality of life, functional outcomes, and possibly long-term recurrence rates. Secondary outcomes might include patient satisfaction, adherence to treatment, and cost-effectiveness.

### Research question:

In individuals with varicose veins & chronic venous insufficiency, how does the integration of emerging technologies and trends with physiotherapy interventions compare to traditional physiotherapy interventions alone in terms of efficacy, as measured by evaluation of venous reflux, volumetry measurement, symptom improvement, quality of life, functional outcomes.

### Inclusion Criteria:

### Study design:

Studies that are entirely experimental, whether randomized or non-randomized/quasi-experimental were included.

### Population:

Research concentrating on people or groups utilizing digital health interventions for their varicose veins.

### Interventions:

This systematic review focuses on articles that assess physiotherapy interventions integrated with emerging technologies for the management of varicose veins. The included studies explored a variety of innovative approaches aimed at enhancing venous return, reducing symptoms, and improving the overall quality of life in individuals with chronic venous insufficiency. Experimental studies with a Pedro score of 7/10 or above were included in the review. Different physiotherapy-based therapies and approaches were used in the studies that were analyzed to assess the results of patients with varicose veins and chronic venous insufficiency (CVI). Graduated compression stockings (GCS), complex decongestive therapy (CDT), intermittent pneumatic compression (IPC), passive ankle movement devices, neuromuscular electrical stimulation (NMES), aquatic therapy (including thermal water-based therapy), nonpneumatic compression devices, adjunctive cryotherapy, and customized graduated elastic compression stockings were among these interventions. Duplex ultrasonography, limb volume and circumference measurements, venous clinical severity scores (VCSS), pain scales, oxygen saturation levels, quality-of-life questionnaires, Doppler studies, and patient-reported outcome measures were among the various assessment methods employed in these studies.

The main objective of the evaluated therapies was to improve venous flow dynamics (using compression devices and stockings), decrease edema and pain (using IPC, CDT, and cryotherapy), and increase venous return by muscle pump activation (e.g., passive ankle motions and NMES). Advanced compression technologies, including compression stockings and nonpneumatic compression devices, aimed to improve comfort, compliance, and treatment efficacy, while other therapies, focused on increasing joint mobility and functional capability. When taken as a whole, these therapies improved clinical outcomes and quality of life by offering conservative, non-invasive options that were customized for each patient.

In terms of improved venous hemodynamics, decreased edema, improved functional outcomes, and symptom reduction, the therapies generally showed significant results. The interactive and accessible character of the technologies has also been linked to higher levels of patient satisfaction and accessibility, according to several studies. Some articles did, however, draw attention to issues with cost-effectiveness, long-term user compliance, and the requirement for additional extensive testing to prove long-term usefulness. The review included peer-reviewed research papers sourced from databases such as PubMed (MEDLINE), Scopus, and Web of Science, with full texts published between January 1, 2018, and March 31, 2025.

### Exclusion Criteria:

### Research Studies:

Studies without original data and non-experimental studies were excluded i.e. editorials, short communications, qualitative studies, case-control studies.

### Irrelevant population:

Studies which were conducted on patients with conditions other than varicose veins or had any other comorbidities.

### Language and Date Restrictions:

Articles not published in English or those outside the predefined date range for the review.

### Availability of Full Protocol:

A full protocol has been written and uploaded to PROSPERO. The protocol will be made available after the review is completed.

### Study risk of bias/Quality assessment:

Risk of bias was assessed using: Cochrane RoB-2, Newcastle-Ottawa and ROBINS-I. Data was assessed independently by at two co-authors. Additional information was sought from study investigators if required information was unclear or unavailable in the study publications/reports.

### Reporting bias assessment:

Risk of bias due to missing results was not assessed.

### Certainty assessment:

This was done using the GRADE (Grading of Recommendations, Assessment, Development, and Evaluations) approach. The GRADE framework evaluates the certainty of evidence for each outcome based on several factors, including study design, risk of bias, consistency, directness, precision, and publication bias.

### Outcomes analyzed:

### Main outcomes:

Primary Outcomes: Symptom improvement, quality of life, functional outcomes, recurrence rates. Secondary Outcomes: Patient satisfaction, adherence to treatment, cost-effectiveness, adverse events.

## RESULTS

A total of **10 studies** were included in this systematic review, evaluating emerging physiotherapeutic and technological interventions for varicose veins and chronic venous insufficiency (CVI).

### Complex Decongestive Therapy (CDT):

One RCT (Jiménez et al., 2025) reported that CDT, which includes manual lymphatic drainage, intermittent pneumatic pressotherapy, and bilayer bandaging, improved symptoms, venous flow, BMI, and quality of life, particularly in the physical domain. Limitations included small sample size and lack of objective edema measurement.

### Compression Devices:

Two studies investigated compression-based interventions. Another study (2024) showed that customized graduated compression stockings improved patient adherence, pressure stability, and quality of life compared with standard stockings. Barfield et al. (2025) demonstrated that nonpneumatic compression devices reduced limb volume, enhanced adherence, and improved quality of life more effectively than advanced pneumatic compression devices.

### Aquatic/Thermal Therapy:

Two studies (Sharifi et al., 2021; Menegatti et al., 2021) found that aquatic therapy, including thermal water exercise, improved venous symptoms, reduced edema, enhanced calf muscle pump function, and improved quality of life. Improvements persisted up to 24 months, though studies were limited by small sample size and short-term follow-up for some outcomes.

### Intermittent Pneumatic Compression (IPC):

Mohamed et al. (2020) reported that IPC significantly reduced ankle girth, improved venous flow, and alleviated pain compared with medical treatment alone.

### Cryotherapy:

One study (Kelechi et al., 2018) found that cryotherapy provided no additional benefit over standard compression and leg elevation for pain reduction or self-efficacy.

**Table-I:** Summary of studies evaluated.

Authors, Year, Ref	Title	Methodology	Results	Conclusion	Limitation	Practical Implications
Wnuk et al, 2024^4^	Effect of passive ankle movement in the sitting position on the symptoms of chronic venous insufficiency with long-term observation	A prospective, controlled experimental study on 58 patients who suffered from chronic venous insufficiency and 37 normal sedentary adults. Participants of study utilized Bella Vena for thirty minutes/day for eight months. Patients were supervised monthly via in follow up sessions as well as remote consultations.	There was a significant decrease in symptoms (p ≤ 0.01), including increased oxygen saturation, decreased venous reflux, improved venous return (Doppler ultrasound), and decreased pain (VAS scale).	The Bella Vena device can effectively improve venous function and lessen the symptoms of CVI by passive ankle movement. It offers a practical, non-invasive substitute for vigorous exercise, particularly for people with limited mobility.	The study was restricted to eight months of observation, lacked a randomized control group, and had a comparatively small sample size. It is still unknown what the long-term impacts and wider population applicability will be.	Bella Vena and other robotic-assisted physiotherapy devices can be included into home-based rehabilitation programs, providing a convenient, non-invasive way to help patients with varicose veins and CVI, especially those who are unable to engage in physical exercises.
Ravikumar et al., 2017^5^	Effectiveness of Footplate Neuromuscular Electrical Stimulation in Enhancing Venous Return in Patients with Chronic Venous Disease: A Randomized Controlled Trial	22 CVD patients participated in a randomized controlled experiment (CEAP C2-C4). Over the course of six weeks, participants were randomized to utilize either a sham device or the REVITIVE IX NMES device.	Significant improvements (p < 0.0001) were observed in the femoral vein peak velocity (+377.7%) and volume flow (+107.9%) in the NMES group. Additionally, the NMES group’s limb volume remained constant while the sham group’s increased. The NMES group’s quality of life scores improved more than the sham group’s.	For patients with chronic venous illness, NMES with the REVITIVE IX device is a successful non-invasive treatment that improves venous return, lowers oedema, and improves quality of life. Its potential as a supplement to conventional physiotherapy is highlighted by the study.	Brief (6-week) intervention period and small sample size. absence of a long-term monitoring program and no evaluation of recurrence rates over time.	In order to enhance venous return and reduce limb edema, NMES devices might be utilized as supplemental tools in physiotherapy, particularly for patients with restricted mobility. Future wearable and AI integration may improve patient adherence and treatment effectiveness even more.
Kakkos et al., 2018^6^	The Effectiveness of Graduated Compression Stockings in Preventing Venous Reflux in Pregnant Women: A Randomized Controlled Trial	Sixty pregnant women in a randomized controlled study, thirty of whom received graduated compression stockings as part of the intervention and thirty of whom did not. Duplex ultrasonography was used to measure venous reflux parameters during the pregnancy.	The intervention group’s great saphenous vein reflux time decreased significantly (p < 0.0001), from 0.13 seconds to 0.04 seconds. The control group’s time increased significantly from 0.02 to 0.34 seconds, demonstrating that their venous insufficiency was progressing. By the conclusion of pregnancy, the intervention group had no pathological reflux.	As a non-invasive physiotherapy-based preventive measure, graduated compression stockings are quite successful in halting the progression of varicose veins and venous reflux during pregnancy.	The study was restricted to a particular pregnancy period and had a small sample size. Long-term postpartum effects and the ideal compression levels for varying severities were not examined.	For pregnant women at risk of varicose veins, compression stockings should be a regular part of their physiotherapy regimen. AI integration in the future for remote monitoring and customized compression may improve results.
Jiménez et al., 2025^7^	Effectiveness of Complex Decongestive Therapy in Chronic Venous Insufficiency: A Randomized Controlled Trial	CDT (manual lymphatic drainage, intermittent pneumatic pressotherapy, and bilayer bandage) was evaluated in patients with CVI in a single-blind, randomized controlled experiment.	There was a notable improvement in QoL (pain, physical, social, and psychological domains) right after therapy; at six weeks, only the physical component was still significant. At six weeks, there was still improvement in venous flow velocity and ISV diameter along with symptom reduction (VCSS). Fat mass and BMI stayed constant and declined. Patients’ perceptions of edema improved, but there was no objective measurement.	For CVI, CDT is a successful conservative treatment that improves symptoms, venous flow, BMI, fat mass, and quality of life. It is a non-invasive, safe choice that may be incorporated into CVI patients’ physiotherapy treatment regimens.	Lack of objective edema measurement, small sample size, and variation in disease severity. To verify long-lasting benefits, longer-term monitoring is required.	One important conservative strategy for treating CVI is CDT. Measurable gains in physical health and quality of life can be obtained by incorporating it into physiotherapy. Objective edema assessments and longer-term follow-up should be the goals of future regimens.
Barfield et al., 2025^8^	TEAYS: A Multicenter Randomized Crossover Trial Comparing Nonpneumatic and Pneumatic Compression Devices for Lower Extremity Lymphedema	71 individuals (108 limbs) with primary or secondary lower extremity lymphedema participated in a multicenter, prospective, randomized crossover study. For ninety days each, each patient employed an advanced pneumatic compression device (APCD) and a nonpneumatic compression device (NPCD), with a washout period in between.	In comparison to APCD, NPCD decreased limb volume more (369.9 mL vs. 83.1 mL; p <.05), enhanced overall quality of life (NPCD significant; APCD not significant), and demonstrated superior adherence (81% vs. 56%; p <.001) and satisfaction (78% vs. 22%). Compared to APCD, 91% of NPCD users were active during treatment.	A promising substitute for APCD in the management of lymphedema, NPCD showed higher efficacy in lowering edema, improving QoL, and increasing treatment adherence and satisfaction.	Short follow-up; crossover design; possible bias in self-reported adherence/QoL; potential effects that persist even after washout.	For patients with lymphedema, NPCD provides a practical, easy-to-use, and more lifestyle-compatible alternative, which supports its inclusion in physiotherapy regimens, particularly when patient adherence and mobility are issues.
Yang et al., 2024^9^	Comparison of Standard vs Customized Graduated Compression Stockings for Chronic Venous Disease: A Randomized Controlled Trial	RCT comparing standard GECSs (s-GECSs) with customized GECSs (c-GECSs) over a 6-month period with 79 CVD patients (CEAP C2-C3).	c-GECSs demonstrated improved compliance (10.7 vs. 9.5 hours/day, p < 0.05), stable pressure, and significant VEINES-QOL improvement at all time points (p < 0.0001). Similar calf volume reduction was seen in both groups; s-GECSs showed less adherence and greater pressure fluctuation.	The use of c-GECSs in the management of varicose vein physiotherapy was supported by their superiority over s-GECSs in terms of enhancing patient compliance, pressure stability, and quality of life.	Limited sample size; no follow-up for more than six months; no severity-based subgroup analysis.	Patient outcomes and adherence are improved by customized compression stockings; future AI-guided fitting and monitoring could further optimize treatment for chronic venous insufficiency.
Sharifi et al., 2021^10^	The ATLANTIS Trial: Effects of Aquatic Therapy on Chronic Venous Insufficiency	Patients n= 201 with advanced CVI participated in an RCT at A.T. Still University in Arizona that contrasted normal care with aquatic therapy, which involves swimming or walking in water, over a 24-month period.	Improvements in venous symptoms, lower limb edema, and quality of life were observed at three months and continued for 24 months after receiving aquatic therapy. The function of the calf muscle pump and venous return were improved by hydrostatic pressure.	For varicose veins and CVI, aquatic therapy is a successful physiotherapy technique that provides long-lasting symptom alleviation and functional enhancement.	Adherence and aquatic intensity were self-reported; there was no subgroup analysis by CVI stage; the design was single-center.	The main treatment for CVI should be aquatic therapy; future technological integration (such as AI exercise monitoring) may improve individualized aquatic therapy regimens for CVI.
Menegatti et al., 2021^11^	The DATA Study: Dryland vs. Thermal Aquatic Standardized Exercise Protocol for Chronic Venous Disease	CEAP class III CVD patients n=34 participated in an RCT in Italy; they were randomized to receive 30-minute sessions, twice a week, for five weeks, either thermal aquatic therapy (TW) or dryland exercise (DL).	The TW group’s QoL scores were higher, their lower limb volume was significantly reduced, and their GSV caliber was improved along with their ankle range of motion. Moderate ROM/QoL improvements and negligible volume/caliber changes were observed in the DL group.	Thermal water therapy is preferable to dryland exercise as a physiotherapeutic intervention because it reduces edema and improves venous return, joint mobility, and quality of life in patients with cardiovascular disease.	Lack of long-term follow-up to evaluate long-term benefits; small sample size; brief duration.	For the management of CVD, aquatic therapy with thermal water should be given priority. Future studies may incorporate AI to personalize aquatic regimens and track progress in real time for improved rehabilitation results.
Mohamed et al., 2020^12^	Intermittent Pneumatic Compression Therapy for Varicose Veins: Effects on Venous Flow, Ankle Girth, and Pain Levels	Women n=40 (CEAP class 2-3) participated in an RCT and were randomized to receive either medical treatment alone (Group B) or IPC therapy (Group A), which involved five sessions per week for eight weeks.	When compared to medical treatment alone, IPC dramatically reduced ankle girth, reduced pain, and improved venous blood flow (increased flow volume and pulsatility index in femoral and popliteal veins) (p < 0.05).	In patients with varicose veins, IPC is a successful, non-invasive adjunct physiotherapy treatment that improves venous circulation, lessens limb edema, and relieves symptoms.	Only women were included in the small sample, and there was no long-term follow-up.	In order to improve venous return and alleviate symptoms, IPC can be suggested as a supportive physiotherapy intervention in the care of varicose veins; further research may optimize the frequency and length of sessions.
Kelechi et al., 2018^13^	Cryotherapy in Chronic Venous Disease: A Randomized Controlled Trial	RCT with 276 participants (C4-C5 CVeD), randomized to receive cooling leg cuff vs. placebo cuff, both alongside compression therapy and leg elevation..	There was no extra benefit from cooling therapy; both groups saw a significant decrease in pain (p < 0.0001). Both groups’ self-efficacy levels did not alter.	In patients with CVeD, standard management (compression and elevation) efficiently lowers pain; cryotherapy offers no further advantages.	Only C4-C5 phases were monitored; there was no long-term follow-up; a placebo cuff would have offered some cooling.	Emphasizes the value of compression and elevation in physical therapy; according to available data, cryotherapy is not advised as an adjunct for varicose vein pain alleviation.

### Summary:

Overall, CDT, customized compression devices, nonpneumatic compression devices, and aquatic/thermal therapy were consistently associated with improved quality of life, reduced pain, and better functional outcomes in patients with varicose veins or CVI. Evidence for cryotherapy was limited, and it did not offer significant additional benefits over standard physiotherapy. Interventions were generally safe and non-invasive, though sample sizes and follow-up duration varied across studies.

## DISCUSSION

The findings of this systematic review align with and expand upon prior research on physiotherapy interventions for varicose veins and chronic venous insufficiency. While earlier studies like those by Patel et al.^14^ established the efficacy of conventional approaches such as neuromuscular electrical stimulation (NMES) and compression therapy, this review demonstrates how emerging technologies like robotic-assisted devices, AI-enhanced NMES, and advanced compression systems offer additional benefits in venous return enhancement and symptom management.

The review corroborates Santler et al. and Goerge et al.^15^ emphasis on compression therapy as foundational, with recent evidence from Kakkos et al. (2018)^6^ and Barfield et al.^8^ (2025) supporting both traditional graduated stockings and innovative nonpneumatic alternatives. However, it diverges from older literature by highlighting the superior outcomes of aquatic therapy (Sharifi et al., 2021; Menegatti et al, 2021)^10^,^11^ and the limitations of cryotherapy (Kelechi et al., 2018),^13^ suggesting a need for more selective modality application.

The findings of this review align with prior studies demonstrating the effectiveness of laser-based interventions for varicose veins. For instance, Liu et al. evaluated endovenous laser treatment (EVLT) in a clinical setting and concluded that EVLT is both safe and effective for reducing symptoms and recurrence in varicose vein patients. Although EVLT is a surgical intervention, its minimally invasive nature and incorporation of laser technology represent a key example of how emerging technologies are being adopted within modern vascular surgical practice, supporting the broader theme of this review.^16^

Notably, the integration of AI-driven personalization and wearable monitoring systems (Sinthia et al., 2024)^3^ represents a significant advancement over conventional methods, addressing long-standing adherence challenges identified in studies like.^17^,^18^ While confirming the value of established interventions, the review underscores how technological innovations are reshaping treatment paradigms, though it also cautions that some newer approaches like cryotherapy may not outperform traditional care.

Future research should focus on large-scale validation of these technologies and development of standardized protocols to fully realize their potential in clinical practice, as suggested by longitudinal studies like Wnuk et al.^4^ (2024) on passive movement therapies. This synthesis of evidence ultimately points toward an evolving model of care that strategically combines proven physiotherapy techniques with targeted technological enhancements for optimal patient outcomes.

## CONCLUSION

This systematic review highlights the Promising yet evolving role of emerging technologies in enhancing physiotherapy-based management of varicose veins. However, the heterogeneity of existing studies, differences in patient demographics, and inconsistent intervention protocols underscore the necessity for more rigorous, standardized comparative trials to establish definitive treatment hierarchies Future research should prioritize high-quality randomized controlled trials with long-term follow-ups to validate the efficacy, safety, and cost-effectiveness of these emerging technologies, ensuring evidence-based integration into clinical practice. Until then, a tailored, patient-centered approach combining traditional physiotherapy with judicious use of advanced technologies remains the most pragmatic strategy for optimizing varicose vein management.

### Medical Subject Headings:

Humans; Physical Therapy Modalities; Quality of Life; Varicose Veins; Massage; Exercise Therapy; Electric Stimulation Therapy; Laser Therapy; Stockings, Compression.

### Check for similar records already in PROSPERO:

PROSPERO identified a number of existing PROSPERO records that were similar to this one (last check made on 6 March 2025). These are shown below along with the reasons given by that the review team for the reviews being different and/or proceeding.

- The reporting and methodology quality of meta-analyses related to interventions about varicose veins: a cross-sectional survey [published 26 March 2019] [CRD42019126722]. The review was judged not to be similar.
- Effectiveness of Advanced Physiotherapy Interventions in Rehabilitating Patients with Neuromyelitis Optica: A Systematic Review [published 24 February 2025] [CRD420250648567]. The review was judged not to be similar.
- Systematic Review on Physiotherapy Interventions for Facial Palsy: A Comprehensive Analysis of Efficacy and Best Practices [published 6 June 2023] [CRD42023430166]. The review was judged not to be similar.
- Systematic Review of Emerging Technological Interventions for Autistic Individuals’ Transition to and Integration in the Workplace [published 3 December 2024] [CRD42024616473]. The review was judged not to be similar.
- Integrated Physiotherapy Approach for Susac Syndrome: A Comprehensive CaseStudy [published 10 April 2024] [CRD42024528739]. The review was judged not to be similar.
- Risk Factors associated with varicose veins: systematic review and meta-analysis [published 26 July 2023] [CRD42023445306]. The review was judged not to be similar.

### Authors’ contributions:

**DA:** Conception of idea, Data extraction.

**SA:** Data extraction, compiling, critical appraisal of review draft. final revisions and will be accountable for integrity of the study.

**SKK, SA and KH:** Were responsible for collecting the data and compiling.

**MNB:** Was the guarantor and has approved the file for submission after critically review.
